# Using phenome-wide association to investigate the function of a schizophrenia risk locus at SLC39A8

**DOI:** 10.1038/s41398-019-0386-9

**Published:** 2019-01-29

**Authors:** Thomas H. McCoy, Amelia M. Pellegrini, Roy H. Perlis

**Affiliations:** Center for Quantitative Health, Division of Clinical Research and Center for Human Genetic Research, Massachusetts General Hospital, Boston, MA 02114 USA

## Abstract

While nearly all common genomic variants associated with schizophrenia have no known function, one corresponds to a missense variant associated with change in efficiency of a metal ion transporter, ZIP8, coded by SLC39A8. This variant has been linked to a range of phenotypes and is believed to be under recent selection pressure, but its impact on health is poorly understood. We sought to understand phenotypic implications of this variant in a large genomic biobank using an unbiased phenome-wide approach. Specifically, we generated 50 topics based on diagnostic codes using latent Dirichlet allocation, and examined them for association with the risk variant. Then, any significant topics were further characterized by examining association with individual diagnostic codes contributing to the topic. Among 50 topics, 1 was associated at an experiment-wide significance threshold (beta = 0.003, uncorrected *p* = 0.00049), comprising predominantly brain-related codes, including intracranial hemorrhage, cerebrovascular disease, and delirium/dementia. These results suggest that a functional variant previously associated with schizophrenia risk also increases liability to cerebrovascular disease. They further illustrate the utility of a topic-based approach to phenome-wide association.

## Introduction

Despite the remarkable success of genome-wide association studies (GWAS) in medicine, a central challenge remains extrapolating from common-variant associations to actionable disease biology^1^. In particular, the polygenicity of most common disorders, and the lack of functional single-nucleotide polymorphisms (SNPs), renders follow-up of GWAS challenging even in cellular or animal models.

As a complement to more traditional efforts at functional genomics using model systems, phenome-wide association, or PheWAS, seeks to understand the implications of a risk variant by characterizing associated phenotypes in vivo^2^. However, such studies carry substantial risk of type 1 error because they typically examine 1000 or more phenotypes. Moreover, many biobanks and registries rely on individual billing or claims codes for which reliability varies substantially^3–6^. To address both of these limitations, we have previously demonstrated that the use of probabilistic topic models, an approach drawn from natural language processing that draws on groups of related diagnostic codes rather than individual codes, provides interpretable dimensionality reduction as well as making efficient use of sparse count data (i.e., the fact that most individuals will not have any given diagnosis)^7,8^.

Here we apply this method to examine phenotypic implications of a recently identified common variant associated with schizophrenia risk^9,10^. Notably, among all of the first 108 loci associated with schizophrenia, only this variant is a nonsynonymous coding SNP, which is functional (i.e., the risk allele is associated with decreased metal ion transport) and common in Northern European populations^11^. However, little is known about its physiologic role, particularly in the context of brain function. Therefore, to better understand this schizophrenia risk locus, we conducted a topic-based PheWAS in a large genomic biobank linked to electronic health records (EHRs) of multiple academic medical centers.

## Materials and methods

### Cohort derivation, genotyping, and quality control

The cohort was derived from the first four genotyping waves of the Partners HealthCare Biobank Initiative^12^, which spans *N* = 20,084 inpatients and outpatients across two large academic medical centers and affiliates (*n* = 4927, 5353, 4784, and 5020). Participants provided written informed consent for EHRs to be analyzed in protocols approved by the Partners HealthCare Institutional Review Board, along with a blood sample for DNA extraction.

After extraction of DNA from buffy coat, samples were genotyped via one of the Illumina Multi-Ethnic Genotyping Arrays, which include content from phase 3 of the 1000 Genomes Project. For details of genotyping, see our prior publication^7^. To address potential batch effects across the four genotyping waves, we cleaned, imputed, and analyzed each one separately. In each wave, participants were included with genotyping call rates >99%, and no related individuals based on identity by descent (defined by pi-hat > 0.25)^13^. From these individuals, genotyped SNPs were retained if call rate was at least 95% and Hardy-Weinberg equilibrium *p* value was >1 × 10^–6^. Genotypes were imputed using the Michigan Imputation Server implementing Minimac3^14–16^ with all population subsets from 1000G Phase 3 v5 as reference panel; haplotypes were phased using SHAPEIT^17^. The SNP of interest here is imputed but with a high degree of confidence (rsq/info = 0.911; avg call = 0.99). Minor allele frequency is 0.079, consistent with other reports in European cohorts^18^.

### Ancestry

To address risk for stratification artifact, each genotyping wave was examined via principal components analysis of linkage-disequilibrium-pruned genotyped SNPs as a measure of population substructure, using the PLINK 1.9 implementation of EIGENSTRAT. HapMap samples of Northern European ancestry were used to confirm location of this population group^19–21^, yielding *n* = 3593, 3327, 3552, and 3105 participants from genotyping waves 1–4, respectively.

### Topic identification

As in our prior work, we identified topics based on the ninth revision of the International Statistical Classification of Diseases (ICD-9) diagnosis codes extracted from each individual’s EHR data, further grouped into top-level PheWAS codes intended to capture clinically meaningful disease categories^22^. We then applied frequency controls to eliminate PheWAS codes occurring in <0.5% of subjects, yielding 480 distinct PheWAS codes. The remaining PheWAS code count by subject matrix was used to fit a latent Dirichlet allocation (LDA) model with 50 topics; the 50 topic count was selected for consistency with our own prior work and in the absence of well-established methods for optimal topic count selection^23^. As we have described^7^, this unsupervised machine learning method treats each subject’s medical record as if it were a document composed of PheWAS codes reflecting a mixture of underlying topics, or disease categories. The LDA model that results reflects a distribution of all PheWAS codes over each topic, although most codes contribute only a trivial amount. The fitted topic model was then used to extract topic membership scores for each subject. Topic modeling used R v3.4.3^24,25^.

### Analysis

Primary analysis examined association between the SNP of interest (rs13107325, at chr4:103188709 in hg19) and each of the 50 topics. Single-locus associations in each genotyping wave were examined individually, and then combined in inverse-variance-weighted fixed-effects meta-analysis in Plink 1.9. Tests for association used linear regression assuming an additive allelic effect treated each topic as a quantitative trait, and adjusted for the first 10 principal components a priori. Secondary analysis examined association between presence/absence of each diagnostic code with loading >0.01 (i.e., 1%) on any topics significant at *p* < 0.001 (i.e., 0.05/50 topics) with the SNP of interest; these analyses were similarly adjusted for principal components, and then for body mass index (BMI) as well.

### Follow-up

To further characterize the risk allele, we examined data from the UK Biobank as analyzed by Neale and colleagues^26^ and presented in the Global Biobank Engine (Global Biobank Engine, Stanford, CA; http://gbe.stanford.edu/). We queried rs13107325 to identify health-care codes most strongly enriched in this cohort, then examined the closest-corresponding ICD-10 codes for each individual PheWAS code in our most strongly associated topic.

## Results

**Fig. 1:**
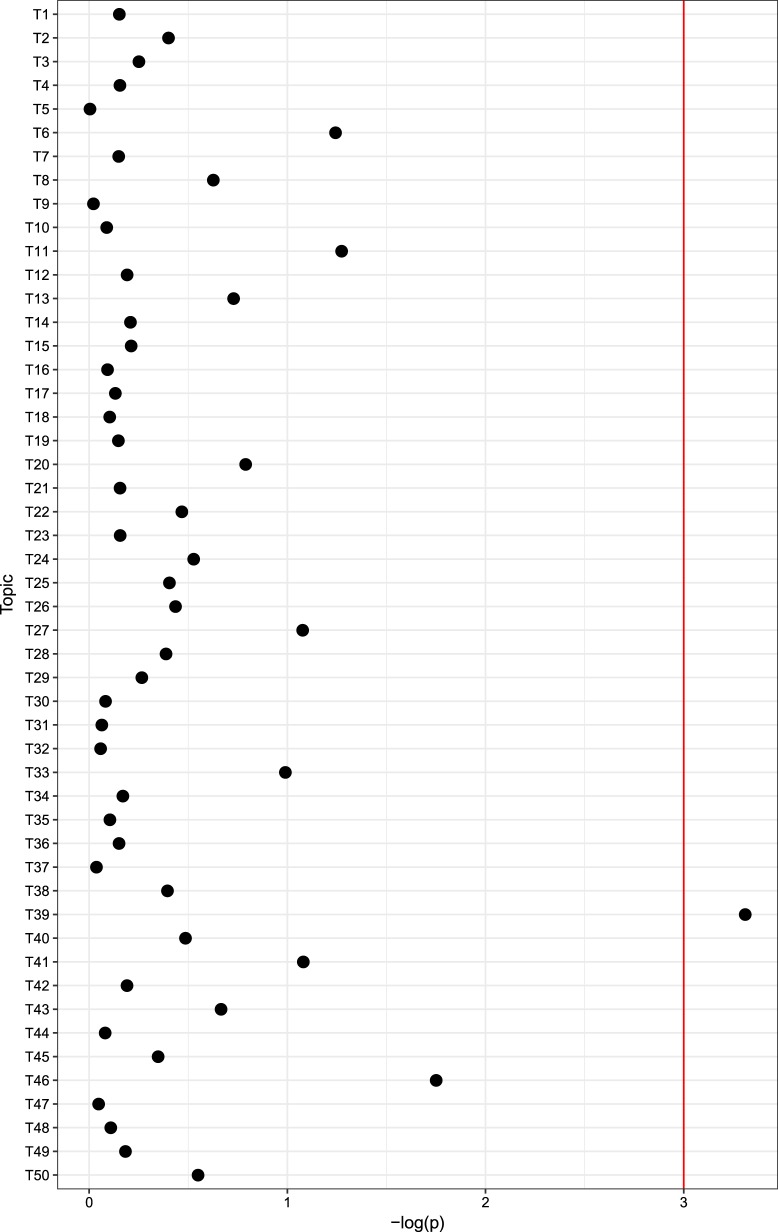
Manhattan plot of association between rs13107325 and individual electronic health record-derived topics. Red line indicates experiment-wide significance

For 13,577 participants, mean age was 60.5 years (SD 16.1), 7473 (55.1%) were female, and mean BMI was 27.6 (SD 6.1). Among the 50 diagnostic topics, 1 was significantly associated with rs13107325 at an experiment-wide significance threshold (*p* = 0.00049; beta = 0.0029 for the minor (schizophrenia risk-increasing) allele) (Fig. 1 and Supplemental Table 1).

We next examined the 13 individual codes loading onto this topic with weights ≥0.01 (i.e., presence of a code is associated with 1% increase in probability of belonging in this diagnostic group). Table 1 lists these codes, along with weight in topic and univariate association. In particular, nominally significant univariate associations were observed with intracranial hemorrhage, delirium/dementia, other conditions of the brain, other cerebral degeneration, vertigo, cerebrovascular disease, and developmental disorders. In all cases, the minor (risk) allele is associated with greater risk for the phenotype. Nominal associations persisted after adjustment for BMI, known to be associated with this SNP in prior genome-wide studies (Table 1)^27^.

**Table 1:** Diagnostic codes with weight in topic and univariate association

Diagnosis	Frequency	*p*	OR	OR (wave 1)	OR (wave 2)	OR (wave 3)	OR (wave 4)	*p* (BMI-adjusted)	OR (BMI-adjusted)	ICD-10	UKBB *p*
Intracranial hemorrhage	0.037	0.0008861	1.4062	1.278	1.851	1.006	1.414	0.004396	1.8189	I61	0.398
Delirium dementia and amnestic disorders	0.080	0.001313	1.269	1.153	1.341	1.274	1.302	0.002229	1.4481	F05	0.913
Other conditions of the brain	0.136	0.008265	1.1737	1.219	1.216	1.136	1.112	0.05571	1.2068	NA	NA
Other cerebral degenerations	0.020	0.012	1.4782	NA	1.548	1.69	1.076	0.6535	0.8377	NA	NA
Vertiginous syndromes	0.245	0.01562	1.127	1.142	1.136	1.109	1.121	0.0179	1.196	H81	0.978
Cerebrovascular disease	0.207	0.02108	1.1293	1.146	1.218	1.106	1.003	0.1055	1.1521	I67	0.584
Developmental delays and disorders	0.022	0.03531	1.4545	1.528	1.389	NA	NA	NA	NA	NA	NA
Neurological disorders due to brain damage	0.154	0.1107	1.0984	1.161	1.153	1.006	1.07				
Abnormal movement	0.157	0.1474	1.0882	1.279	1.033	1.046	0.9888				
Dysphagia	0.112	0.2665	1.0782	0.9607	0.8365	1.256	1.255				
Other disorders of circulatory system	0.086	0.2885	1.0836	0.9709	0.991	1.021	1.454				
Visual disturbances	0.076	0.3032	1.0866	0.8431	1.415	0.9922	1.041				
Hemiplegia	0.033	0.7611	1.0369	0.9339	1.072	0.9803	1.147				

Frequency refers to the proportion of individuals across the four study waves with at least one diagnostic code

*OR* odds ratio, *BMI* body mass index, *NA* frequency too low for regression result, *ICD-10* 10th revision of the International Statistical Classification of Diseases, *UKBB* United Kingdom Biobank

For comparison, we examined ICD-10 diagnostic codes associated with the risk allele by querying the UK Biobank association results using the Global Biobank Engine. For nominal *p* < 1e-03 (the same threshold as applied to our experiment-wide topics), associated diagnoses with increased risk included osteoarthritis (*p* = 2.06e-11), other joint disorder (2.50e-06), arthritis not otherwise specified (1.12e-05), hiatus hernia (1.47e-05), incontinence (2.14e-05), gastroesophageal reflux (3.58e-05), hay fever/rhinitis (8.03e-05), asthma (1.64e-04), motor neuron disease (4.75e-04), joint pain (5.53e-04), and back pain (6.42e-04). Table 1 also reports associations for the closest ICD-10 code corresponding to the individual PheWAS codes in the primary (intracranial hemorrhage-plus) topic; none of these was nominally associated. Notably, however, no association with schizophrenia codes was identified in either the UK Biobank (via ICD-10) (*p* = 0.39) or the Partners HealthCare Biobank (*p* = 0.96).

## Discussion

In this analysis of 13,577 individuals of Northern European ancestry in a large hospital-based biobank linked to EHR, we identified a constellation of diagnostic codes associated with a previously reported schizophrenia risk-associated missense variant. The topic is notable for its coherence—i.e., the extent to which nearly all of the associated codes reflect cerebrovascular disease or sequelae—although some of the associated codes (e.g., “other conditions of the brain”) would not necessarily have been identified a priori as informative for analysis.

Previous work has suggested that this schizophrenia risk SNP is pleiotropic, associated with multiple genome-wide association phenotypes including BMI^27^. Further, some evidence suggests this locus to be under selection pressure^28,29^. Here we sought to identify a group of codes associated with the variant as a means of better understanding potential pathophysiologic mechanisms.

In particular, while the proximal function of the SLC39A8 gene product, ZIP8, is known, the implications of the schizophrenia risk gene are not. Studies of null mutations in rodents suggested important developmental effects^30^, while other functional mutations in humans have been associated with disorders of glycosylation^31^. However, the mechanism by which ZIP8 may contribute to schizophrenia risk is unknown. Speculative mechanisms range from immune modulation to metabolic effects and modulation of excitotoxicity via glutamate signaling (for a review, see Costas)^11^. Our results are consistent with both of these, and suggest the utility of investigating the transporter further in stroke-related injury associated with immune activation, where levels of ZIP8 expression have been shown to be high^32^.

While we were unable to directly examine PheWAS/ICD-9 code-based topics in the UK Biobank, we did seek to examine the nearest match in individual ICD-10 codes. This analysis does not represent replication per se, as the correspondence between ICD-9 and -10 codes may be poor. Among those ICD-10 codes significantly increased in individuals with the risk allele, the preponderance relate to osteoarthritis and joint pain, which may be sequelae of obesity. Notably, we do not find evidence of replication for individual ICD-9 code associations mapped to ICD-10—nor even of replication of the robust schizophrenia association reported in prior studies (*p* = 1.54e-12; odds ratio 1.16, SE 0.02). Taken together, these follow-up results underscore the challenges in using single diagnostic codes, particularly when comparing across health systems. They further illustrate the need for additional replication of our topic-based approach.

Nonetheless, our results provide further support for the notion that topic-based genome-wide association is a powerful means of addressing the variable reliability of individual diagnostic codes while facilitating phenome-wide investigation, or simply reverse genomics^7^. It provides control of type I error by limiting the number of phenotypes tested, such that only topics achieving experiment-wide association require further investigation. In prior work, we demonstrated that under most scenarios, power to detect association will be greater with this approach; the exception is circumstances where a single diagnostic code captures essentially all of the relevant variance associated with a variant.

The extrapolation from GWAS results to biology remains a great challenge, particularly for brain diseases where model systems may be more limited. Nonetheless, if the promise of modern genomics is to be fulfilled, bridging this gap is necessary, particularly to enable development of pharmacologic interventions tied to genomics as has been done in other disorders^1^. For schizophrenia, investigating the ZIP8 locus in large biobanks may help to complement and extend efforts in cellular and animal models to understand this complex disease.

## Disclaimer

The sponsor had no role in study design, writing of the report, or data collection, analysis, or interpretation. The corresponding and senior authors had full access to all data and made the decision to submit for publication.

## Supplementary information

Supplementary Information
