# Supplementary material for: Using phenome-wide association to investigate the function of a schizophrenia risk locus at SLC39A8
Source: Transl Psychiatry. 2019 Jan 29;9:45. doi: 10.1038/s41398-019-0386-9 (PMC6351652; doi:10.1038/s41398-019-0386-9)
Supplement: Supplementary file 1 — Supplementary Information [file 41398_2019_386_MOESM1_ESM.docx]

**2018TP000310RR**

**Title:** Using phenome-wide association to investigate the function of a schizophrenia risk locus at SLC39A8

**Authors:** Thomas H. McCoy, Jr MD, Amelia M. Pellegrini BA, Roy H. Perlis MD MSc

**Supplemental Material:**

**Supplemental Table 1.** Topic associations by genotyping wave

**Supplemental Table 1.** Topic associations by genotyping wave

| Topic | N waves | P | beta | Q | I | beta wave 1 | beta wave 2 | beta wave 3 | beta wave 4 |
| --- | --- | --- | --- | --- | --- | --- | --- | --- | --- |
| T39 | 4 | 0.0004892 | 0.0029 | 0.69 | 0 | 0.0038 | 0.0043 | 0.0016 | 0.0025 |
| T46 | 4 | 0.01774 | 0.0018 | 0.2417 | 28.4 | 0.0003 | 0.0012 | 0.0013 | 0.0045 |
| T11 | 4 | 0.05322 | -0.002 | 0.647 | 0 | -0.0016 | -0.0044 | -0.0017 | -0.0003 |
| T6 | 4 | 0.05712 | -0.0013 | 0.9637 | 0 | -0.0008 | -0.0017 | -0.0016 | -0.0011 |
| T41 | 4 | 0.08304 | -0.0013 | 0.7194 | 0 | -0.0016 | -0.0025 | -0.0014 | 0 |
| T27 | 4 | 0.08369 | -0.0017 | 0.9787 | 0 | -0.0022 | -0.0009 | -0.002 | -0.0015 |
| T33 | 4 | 0.1023 | -0.0016 | 0.6983 | 0 | -0.0008 | -0.0031 | -0.0018 | -0.0005 |
| T20 | 4 | 0.1623 | -0.0007 | 0.3298 | 12.57 | -0.0014 | -0.0004 | 0.0009 | -0.0016 |
| T13 | 4 | 0.1867 | 0.0017 | 0.1909 | 36.87 | 0.0064 | 0.0029 | -0.0024 | 0.0007 |
| T43 | 4 | 0.216 | -0.0007 | 0.5864 | 0 | -0.0016 | -0.0006 | -0.0011 | 0.0006 |
| T8 | 4 | 0.2363 | 0.0008 | 0.4459 | 0 | -0.0007 | 0.0015 | 0.0019 | 0.001 |
| T50 | 4 | 0.2822 | -0.0012 | 0.9443 | 0 | -0.0002 | -0.0006 | -0.0018 | -0.0018 |
| T24 | 4 | 0.2969 | 0.0007 | 0.5265 | 0 | -0.0002 | -0.0004 | 0.0018 | 0.0019 |
| T40 | 4 | 0.3265 | -0.0012 | 0.5928 | 0 | -0.0021 | 0.0005 | 0.0002 | -0.004 |
| T22 | 4 | 0.3407 | 0.0008 | 0.0596 | 59.57 | -0.0013 | -0.0015 | 0.0034 | 0.0029 |
| T26 | 4 | 0.3666 | -0.0008 | 0.4841 | 0 | -0.0005 | -0.0023 | 0.0011 | -0.0016 |
| T25 | 4 | 0.3935 | 0.0005 | 0.6252 | 0 | -0.0001 | 0.0016 | 0.0007 | -0.0004 |
| T2 | 4 | 0.3977 | 0.0008 | 0.1962 | 35.99 | 0.0034 | 0.0024 | -0.0019 | 0.0009 |
| T38 | 4 | 0.4026 | 0.0006 | 0.0089 | 74.11 | -0.0012 | 0.0039 | -0.002 | 0.002 |
| T28 | 4 | 0.4094 | -0.0007 | 0.7582 | 0 | -0.0004 | 0.0006 | -0.0017 | -0.0013 |
| T45 | 4 | 0.4487 | -0.0005 | 0.7877 | 0 | -0.0011 | 0.0002 | 0.0001 | -0.0015 |
| T29 | 4 | 0.5423 | -0.0004 | 0.7342 | 0 | -0.0003 | -0.0011 | -0.001 | 0.0006 |
| T3 | 4 | 0.561 | 0.0005 | 0.5603 | 0 | -0.0009 | 0.0009 | 0.0022 | 0.0008 |
| T15 | 4 | 0.6139 | -0.0003 | 0.8476 | 0 | -0.0011 | 0.0005 | -0.0003 | -0.0004 |
| T14 | 4 | 0.6189 | 0.0004 | 0.0559 | 60.36 | 0.0047 | -0.0026 | 0.0011 | 0.0007 |
| T12 | 4 | 0.6435 | 0.0004 | 0.2139 | 33.07 | -0.0027 | 0.0031 | 0.0005 | 0.001 |
| T42 | 4 | 0.6442 | -0.0004 | 0.4775 | 0 | -0.0019 | 0.0029 | -0.0016 | 0.0002 |
| T49 | 4 | 0.6558 | -0.0003 | 0.8786 | 0 | 0.0002 | -0.001 | -0.0003 | 0.0004 |
| T34 | 4 | 0.676 | -0.0003 | 0.9998 | 0 | -0.0002 | -0.0003 | -0.0003 | -0.0004 |
| T23 | 4 | 0.6975 | 0.0003 | 0.1319 | 46.58 | 0.0013 | 0.0035 | -0.0016 | -0.0006 |
| T21 | 4 | 0.6986 | -0.0004 | 0.6221 | 0 | -0.0017 | 0.002 | -0.0013 | 0.001 |
| T4 | 4 | 0.6995 | 0.0004 | 0.6124 | 0 | 0.0025 | -0.0001 | -0.001 | 0.0002 |
| T1 | 4 | 0.7044 | 0.0003 | 0.934 | 0 | 0.0004 | -0.0005 | 0.0011 | 0.0001 |
| T36 | 4 | 0.7067 | 0.0002 | 0.256 | 25.94 | 0.0016 | -0.0019 | 0.0007 | 0.0007 |
| T7 | 4 | 0.7094 | -0.0003 | 0.1128 | 49.8 | 0.0023 | 0.0006 | -0.0017 | -0.0025 |
| T19 | 4 | 0.7121 | -0.0003 | 0.653 | 0 | -0.0019 | 0.0002 | 0.0008 | -0.0006 |
| T17 | 4 | 0.7376 | 0.0003 | 0.7332 | 0 | -0.0017 | 0.0008 | 0.0003 | 0.0017 |
| T48 | 4 | 0.7779 | -0.0002 | 0.2096 | 33.78 | -0.0028 | -0.0002 | 0.0018 | 0.0004 |
| T35 | 4 | 0.7858 | -0.0002 | 0.219 | 32.21 | 0.0015 | -0.0016 | -0.0004 | 0.0008 |
| T18 | 4 | 0.7876 | -0.0002 | 0.0652 | 58.44 | 0.0023 | -0.0035 | 0.0016 | -0.0007 |
| T16 | 4 | 0.8077 | -0.0001 | 0.2855 | 20.76 | 0.0016 | -0.0004 | -0.0005 | -0.0017 |
| T10 | 4 | 0.8155 | 0.0002 | 0.359 | 6.83 | 0.0003 | -0.0018 | 0.0024 | -0.0001 |
| T30 | 4 | 0.8268 | -0.0001 | 0.2156 | 32.79 | -0.001 | 0.0015 | -0.0004 | -0.0002 |
| T44 | 4 | 0.8304 | 0.0002 | 0.6817 | 0 | 0.0013 | -0.0018 | 0.0009 | -0.0001 |
| T31 | 4 | 0.8633 | -0.0001 | 0.959 | 0 | -0.0005 | -0.0002 | -0.0001 | 0.0006 |
| T32 | 4 | 0.8755 | -0.0001 | 0.6002 | 0 | -0.0002 | -0.0013 | 0.0015 | -0.0007 |
| T47 | 4 | 0.8958 | 0.0001 | 0.0954 | 52.81 | 0.0025 | -0.0012 | 0.0003 | -0.0022 |
| T37 | 4 | 0.9187 | 0.0001 | 0.7207 | 0 | 0 | -0.0008 | 0.0016 | -0.0006 |
| T9 | 4 | 0.9513 | 0 | 0.111 | 50.1 | -0.0021 | 0.0006 | 0 | 0.0036 |
| T5 | 4 | 0.9904 | 0 | 0.0178 | 70.29 | 0.0005 | 0.0034 | 0.0004 | -0.0035 |
|  |  |  |  |  |  |  |  |  |  |
| Q and I refer to measures of heterogeneity | | | | |  |  |  |  |  |
